# COVID-19 outbreaks among crew members in non-cruise vessels anchoring in Salvador, Brazil, 2021

**DOI:** 10.1590/0074-02760220114

**Published:** 2022-11-14

**Authors:** Cristiane Wanderley Cardoso, Mirela Maisa da Silva Souza, Ana Claudia Venegeroles de Sá Teles, Hernan Dario Argibay, Olivete Borba dos Reis, Felicidade Mota Pereira, Marta Giovanetti, Tereza Magalhaes, Guilherme Sousa Ribeiro

**Affiliations:** 1 Secretaria Municipal de Saúde de Salvador, Salvador, BA, Brasil; 2 Fundação Oswaldo Cruz-Fiocruz, Instituto Gonçalo Moniz, Salvador, BA, Brasil; 3 Agência Nacional de Vigilância Sanitária, Salvador, BA, Brasil; 4 Secretaria de Saúde do Estado da Bahia, Salvador, BA, Brasil; 5 Fundação Oswaldo Cruz-Fiocruz, Instituto Oswaldo Cruz, Laboratório de Flavivírus, Rio de Janeiro, RJ, Brasil; 6 Universidade Federal da Bahia, Faculdade de Medicina, Salvador, BA, Brasil; 7 Colorado State University, Center for Vector-Borne Infectious Diseases, Department of Microbiology, Immunology and Pathology, Fort Collins, CO, USA

**Keywords:** COVID-19, outbreak, public health, maritime traffic, epidemiology

## Abstract

**BACKGROUND:**

The coronavirus disease 2019 (COVID-19) pandemic, caused by the severe acute respiratory syndrome coronavirus 2 (SARS-CoV-2), has affected the maritime sector due to virus transmission onboard and traffic restrictions. However, reports of SARS-CoV-2 transmission on board have been mostly restricted to those occurring on cruise ships.

**OBJECTIVES:**

To report COVID-19 outbreaks in eight non-cruise vessels and discuss measures to prevent and control the onboard transmission of SARS-CoV-2.

**METHODS:**

We investigated outbreaks of COVID-19 on vessels anchoring in Baía de Todos-os-Santos, Salvador, Brazil, between February and November 2021.

**FINDINGS:**

Most vessels were cargo ships that had docked several times before anchoring in Salvador (five had docked in ≥ 9 ports). The crew ranged from 22 to 63 members. The infection attack rate on each vessel ranged from 9.7 to 88.9%. The risk of symptomatic infection largely varied among the crew of each vessel (0 to 91.6%). Overall, the risk of developing COVID-19 signs and symptoms was lower among crew members vaccinated (age-adjusted risk ratio: 0.19; 95% confidence interval 0.06-0.65). SARS-CoV-2 variants not previously identified in Salvador were detected (C.14, B.1.617.2 and B.1.351).

**MAIN CONCLUSIONS:**

Despite maritime guidelines to avert COVID-19 on board, outbreaks have happened. The multiple stopovers of non-cruise vessels during their routes may contribute to the spread of SARS-CoV-2 variants worldwide. Reducing the onboard transmission of SARS-CoV-2 depends on joint efforts by the crew and local health authorities and, equally important, achieving high vaccination coverage to prevent infections and illness.

The coronavirus disease 2019 (COVID-19), caused by the severe acute respiratory syndrome coronavirus 2 (SARS-CoV-2) and characterised as a pandemic by the World Health Organization (WHO), has largely impacted maritime traffic and the lives of seafarers and passengers.^1^ ^,^ ^2^ At sea, some of the challenges brought by the pandemic involve the management of cases to prevent additional onboard infections and secondary community infections, crew changes, access to health care in ports, increase in mental health suffering on board and economic losses.^2^

To cope with these challenges and minimise disruptions within the maritime trade, organisations and regulatory agencies have developed recommendations and regulations that shipping companies, crews, port staff and authorities, and local and national governments should adopt.^1^ ^,^ ^3^ ^-^ ^6^ However, the complexity and rapid changes in the epidemiological scenario of SARS-CoV-2 transmission and the constant production of technical/scientific knowledge on the subject have hampered the timely implementation of these actions.^5^ ^,^ ^6^

Maritime traffic refers to all ships and vessels traveling the seas, whether passenger, non-passenger, or mixed. Non-passenger vessels can be of several sorts (cargo, tankers, fishing, among others) and are responsible for over 80% of world trade, playing a vital role in a globalised world and economy.^1^

To date, reports of COVID-19 outbreaks in vessels have been mostly on cruise ships (most notably the outbreak on the Diamond Princess cruise).^7^ ^-^ ^13^ Although scarce,^14^ ^,^ ^15^ reporting outbreaks on non-cruise vessels is of utmost importance for continuously assessing the pandemic’s impacts on maritime trade. In addition, it can help improve integrated domestic and overseas surveillance and containment strategies and regulations, as well as the development of adequate port infrastructure and networks to manage health threats.

Here, we report eight outbreaks of SARS-CoV-2 on different types of non-cruise vessels anchored in Salvador, state of Bahia, northeastern Brazil, in 2021. The port of Salvador is among the 15 most important ports in Brazil (out of 99 coastal ports). In 2020, it received 624 vessels; in 2021, it received 594 vessels.^16^ We also discuss the control actions implemented by the vessels and local and national authorities and the associated challenges.

## METHODS

*Outbreak investigations* - From February to November 2021, the Centro de Informações Estratégicas de Vigilância em Saúde (CIEVS) of Salvador was informed by the Agência Nacional de Vigilância Sanitária (ANVISA) about suspected cases of COVID-19 in eight vessels anchoring in the port of Baía de Todos-os-Santos, Salvador (Table I). The city has one of the longest coastline in Brazil, located along the coast of the country’s Northeast Region. The city has the longest coastline in Brazil, with 80 km bordering the Atlantic Ocean. In addition, it has the largest bay in the country, named Baía de Todos-os-Santos. 

Since the revision of the International Health Regulations (IHR) in 2005,^17^ CIEVS has been the operational unit responsible for detecting and organising the response to events with the potential to constitute a public health emergency in Salvador. ANVISA is the national body responsible for protecting the population’s health through sanitary control of production and consumption of products and services and control of ports, airports and national borders. As established by the national protocol for detection and treatment of suspected cases of COVID-19 in ports, airports and borders,^18^ once ANVISA is communicated of suspected COVID-19 cases on board, it should notify maritime authorities, which in turn designate the port where the crew of the ship should stay in quarantine. As part of CIEVS and ANVISA routine health surveillance activities in ports, recently adapted for handling onboard suspected cases of COVID-19,^6^ ^,^ ^16^ ^,^ ^18^ ^,^ ^19^ they started an investigation on each non-cruise vessel determined to anchor in the Baía de Todos-os-Santos due to a suspect case of SARS-CoV-2 infection.

Promptly after the first notification of suspected cases of COVID-19 on each vessel, staff from CIEVS and the local municipal laboratory visited the quarantined vessels in a speedboat provided by ANVISA (journey duration: 50 minutes - one way) to interview the crew members (CM) and to collect nasopharyngeal swabs and blood samples from every CM for COVID-19 testing. A standard questionnaire was used to assess CM’s demographic and clinical features and to gather information about the vessels. Data on prior vaccination against COVID-19 were also obtained and used to estimate vaccine effectiveness in preventing symptomatic illness among those infected. 

The following public health measures were also implemented to prevent SARS-CoV-2 spread: isolation in specific areas of the vessels of confirmed and suspected cases and of cases’ close contacts, disinfection of surfaces, appropriate handling of clothes used in quarantine zones, daily crew monitoring of signs and symptoms and communication with the crew on the execution of mandatory control measures.^6^

The collected samples (nasopharyngeal swab and/or blood) underwent SARS-CoV-2 testing through reverse transcription-polymerase chain reaction (RT-PCR) and serology (Panbio^TM^ rapid diagnostic test for IgM or IgG against SARS-CoV-2). SARS-CoV-2 infection was defined based on positive RT-PCR or IgM results, regardless of symptoms. In addition, CMs were also considered to have SARS-CoV-2 infection when they had respiratory symptoms and a chest computed tomography scan with findings suggestive of COVID-19 (ground-glass opacity in the lung parenchyma). The main signs and symptoms of COVID-19, such as fever, cough, headache, dyspnoea, ageusia and anosmia, were monitored daily by the vessels’ medical staff and captains and reported to ANVISA and CIEVS. 

*Statistical analysis* - We used absolute and relative frequencies, or medians and ranges, to characterise each vessel’s crew according to demographics, presence of comorbidities, clinical manifestations and outcomes of COVID-19 and laboratory test results. Attack rates for SARS-CoV-2 infection were calculated by dividing the number of subjects who fulfilled the definition for a case of infection by the total number of CM in each vessel multiplied by 100. Among those with SARS-CoV-2 infection, we calculated the risk of developing signs and symptoms of COVID-19 by dividing the number of infected subjects who reported symptoms by the total number of subjects with laboratory evidence of SARS-CoV-2 infection, multiplied by 100. We used Poisson regression analysis with robust variance to estimate the crude relative risk of developing a symptomatic infection among those infected by SARS-CoV-2, according to COVID-19 vaccination status. A similar model was then built adjusting for age as a covariate; we did not adjust the relative risk for sex and comorbidities because all infected CM were males and < 5% had comorbidities. The effectiveness of COVID-19 vaccination in preventing disease among those infected was estimated by subtracting the relative risk for symptomatic infection (after adjusting for age) from one. We performed data analysis using Stata version 14 software (StataCorp, https://www.stata.com).^20^

*Ethics statement* - This manuscript was prepared with secondary data obtained by CIEVS and ANVISA during extensive investigations of COVID-19 outbreaks in vessels quarantined in Baía de Todos-os-Santos. The CM from all the investigated vessels provided verbal consent before the interview. The data analysis was performed using a de-identified anonymous database with clinical and laboratory data obtained from interviews and medical records. To do that, each CM was assigned a unique identification number and personal data, such as names and dates of birth, were not included in the analysed database. COVID-19 reporting is mandatory in Brazil, and surveillance services, such as CIEVS and ANVISA, have access to these data and routinely produce case data reports. The investigations carried out by CIEVS prioritise a quick response to guide the organisation of health services and prevent new cases and future outbreaks. Although CIEVS’s main objective is not to perform scientific research, it has an ethical obligation to disclose to the scientific community, health professionals and public health agents the findings of its investigations whenever they have the potential to guide future research, patient care and surveillance and public health response in Brazil and elsewhere.

## RESULTS

*Overall description of the vessel outbreaks* - The eight vessels with COVID-19 outbreaks had different types of cargo and crews ranging from 22 to 63 members (Table I). The attack rate of SARS-CoV-2 infection, frequency of symptomatic infection and history of COVID-19 vaccination varied widely among each vessel crew (Table II). The attack rate of SARS-CoV-2 infection among all the vessels ranged from 9.7 to 88.9% and the rate of symptomatic infections (among those infected) ranged from 0 to 91.6%. As of mid-May 2021, none of the CM of the first four vessels investigated had received any dose of a COVID-19 vaccine. However, the latter four investigated vessels, which anchored in May, July, August and November, 2021 had 3.7, 40.7, 80.6 and 58.3% of CM vaccinated, respectively. Interestingly, the lowest attack rate and the lowest frequency of symptomatic infection occurred in vessel 7, which had the highest vaccine coverage for COVID-19 (Table II). Overall, the risk of a SARS-CoV-2-infected CM developing signs and symptoms associated with COVID-19 was significantly lower among those vaccinated compared to those who were not [crude risk ratio (RR): 0.21; 95% confidence interval (95%CI) 0.06-0.75; age-adjusted RR: 0.19; 95%CI 0.06-0.65]; the effectiveness against symptomatic disease among those infected was 80.1% after adjusting for age. Details about each outbreak are described below. 

**TABLE I:** Features of vessels anchored in Salvador, Brazil, from January to November 2021, with onboard outbreaks of coronavirus disease (COVID-19)

Outbreak	Type	Flag	Crew members (n)	Port of calls before anchoring in Salvador (n)	Last port of call before anchoring in Salvador (city, country)	Planned^*a*^ final destination (city, country)	Date of anchoring in Salvador	Days anchored in Salvador (under quarantine) (n)
1	Fishing	United Kingdom	63	0	Vigo, Spain	Falkland Islands	January 27, 2021	24
2	Bulk carrier	Malta	22	10	São Luís, Brazil	Vitoria, Brazil	March 31, 2021	11
3	LPG tanker^*b*^	Brazil	26	1	Suape, Brazil	Suape, Brazil	April 11, 2021	16
4	Bulk carrier	Panama	22	10	Suape, Brazil	Paranaguá, Brazil	May 2, 2021	10
5	Oil/chemical tanker	Liberia	27	9	Vila do Conde, Brazil	Aracaju, Brazil	May 27, 2021	25
6	Oil tanker	Brazil	27	10	Angra dos Reis, Brazil	Algeciras, Spain	July 12, 2021	14
7	LNG tanker^*c*^	Denmark	31	9	Corpus Christi, USA	Corpus Christi, USA	August 8, 2021	14
8	Ro-Ro cargo^*d*^	Saudi Arabia	24	3	Durban, South Africa	Durban, South Africa	November 11, 2021	24

*a*: planned final destination when the trip started; *b*: LPG (Liquefied Petroleum Gas); *c*: LNG (Liquefied Natural Gas); *d*: Ro-Ro: Roll on-Roll off cargo ship.

**TABLE II:** Demographic and clinical characteristics of crew members during COVID-19 outbreaks in vessels anchored in Salvador, Brazil, from January to November 2021

Feature	Outbreak 1 (n = 63)	Outbreak 2 (n = 22)	Outbreak 3 (n = 26)	Outbreak 4 (n = 22)	Outbreak 5 (n = 27)	Outbreak 6 (n = 27)	Outbreak 7 (n = 31)	Outbreak 8 (n = 24)
	Number/response (%) or median (min-max)							
Crew characteristics								
Age, years	35 (22-55)	40 (25-67)	38 (22-66)	36 (21-55)	34 (25-59)	39 (22-64)	33 (23-53)	38 (24-56)
Male population	62/63 (98.4)	22/22 (100.0)	23/26 (88.5)	22/22 (100.0)	27/27 (100.0)	25/27 (92.6)	31/31 (100.0)	24/24 (100.0)
Comorbidities^*a*^	1/63 (1.6)	1/22 (4.5)	2/26 (7.7)	4/22 (18.2)	1/27 (3.7)	1/27 (3.7)	0/31 (0.0)	1/24 (4.2)
Confirmed cases of SARS-CoV-2 infection^*b*^	38/63 (60.3)	7/22 (31.8)	22/26 (84.6)	4/22 (18.2)^*c*^	24/27 (88.9)	6/27 (22.2)	3/31 (9.7)	10/24 (41.7)
Laboratory test results for SARS-CoV-2^*d*^								
RT-PCR positive	35/63 (55.6)	6/22 (27.3)	22/26 (84.6)	2/22 (9.1)	24/27 (88.9)	6/27 (22.2)	3/31 (9.7)	10/24 (41.7)
IgM positive	10/62 (15.9)	1/9 (11.1)	1/25 (4.0)	NA	NA	NA	NA	NA
IgG positive	34/62 (54.8)	1/9 (11.1)	3/25 (12.0)	NA	NA	NA	NA	NA
Frequency of symptomatic infection	29/38 (76.3)	4/7 (57.1)	15/22 (68.2)	2/4 (50.0)	22/24 (91.6)	3/6 (50.0)	0/3 (0.0)	2/10 (20.0)
Status of vaccination against COVID-19^*e*^	0/63 (0.0)	0/22 (0.0)	0/26 (0.0)	0/22 (0.0)	1/27 (3.7)	11/27 (40.7)	25/31 (80.6)	14/24 (58.3)
Clinical manifestations among those with symptomatic SARS-CoV-2 infection								
Headache	14/29 (48.3)	2/4 (50.0)	8/15 (53.3)	0/2 (0.0)	12/22 (54.5)	0/3 (0.0)	0/0 (0.0)	0/2 (0.0)
Dyspnoea	7/29 (24.1)	1/4 (25.0)	2/15 (13.3)	2/2 (100.0)	6/22 (27.3)	1/3 (33.3)	0/0 (0.0)	1/2 (50.0)
Fever	5/29 (17.2)	2/4 (50.0)	6/15 (40.0)	1/2 (50.0)	19/22 (86.4)	1/3 (33.3)	0/0 (0.0)	1/2 (50.0)
Cough	15/29 (51.7)	3/4 (75.0)	8/15 (53.3)	1/2 (50.0)	19/22 (86.4)	2/3 (66.6)	0/0 (0.0)	1/2 (50.0)
Anosmia/ageusia	7/29 (24.1)	0/4 (0.0)	5/15 (33.3)	0/2 (0.0)	12/22 (54.5)	1/3 (33.3)	0/0 (0.0)	1/2 (50.0)
Odynophagia	11/29 (37.9)	2/4 (50.0)	6/15 (40.0)	0/2 (0.0)	13/22 (59.1)	0/3 (0.0)	0/0 (0.0)	1/2 (50.0)
Disease outcomes^*f*^								
Hospitalisation	7/29 (24.1)	3/4 (75.0)	2/15 (13.3)	2/2 (100.0)	5/22 (22.7)	1/3 (33.3)	0/0 (0.0)	1/2 (50.0)
Days between symptoms onset and hospitalisation	8 (6-10)	1 (0-1)	3 (1-5)	4 (3-5)	5 (2-10)	-	0/0 (0.0)	-
Days of hospitalisation	4 (2-25)	5 (3-5)	8.5 (4-24)	3 (2-4)	4 (1-4)	29	0/0 (0.0)	-
O_2_ supply	3/7 (42.9)	0/3 (0.0)	2/2 (100.0)	1/2 (50.0)	3/5 (60.0)	1/1 (100.0)	0/0 (0.0)	1/1 (100.0)
Intensive care unit admission	3/7 (42.9)	0/3 (0.0)	0/2 (0.0)	0/2 (0.0)	1/5 (20.0)	1/1 (100.0)	0/0 (0.0)	1/1 100.0)
Mechanical respiratory support	3/7 (42.9)	0/3 (0.0)	0/2 (0.0)	0/2 (0.0)	0/5 (0.0)	1/1 (100.0)	0/0 (0.0)	0/1 (0.0)
Death (among those with symptoms)	0 (0.0)	0 (0.0)	0 (0.0)	0 (0.0)	0 (0.0)	1/3 (33.3)	0/0 (0.0)	0/2 (0.0)
SARS-CoV-2 variant detected (no. of samples)	C.14 variant (2)	VOC P.1 (6)	NA	NA	NA	VOC B.1.617.2 (1)	VOC B.1.351 (1)	VOC B.1.617.2 (4)

*a*: comorbidities included obesity (two individuals), diabetes *mellitus* (two individuals), asthma (three individuals) and systemic arterial hypertension (four individuals); *b*: confirmation of severe acute respiratory syndrome coronavirus 2 (SARS-CoV-2) infection was based on a positive reverse transcription-polymerase chain reaction (RT-PCR) result, a positive IgM result or the presence of compatible clinical symptoms accompanied by a chest tomography suggestive of SARS-CoV-2 infection; *c*: include two patients diagnosed solely based on compatible clinical symptoms and chest tomography findings; COVID-19: coronavirus disease 2019; *d:* some patients were tested serially by more than one type of test and thus they may have been positive on more than one test; *e*: use of at least one dose of a vaccine against SARS-CoV-2; *f*: frequency of disease outcomes among those hospitalised, except for death, which is shown for those with symptoms; NA: not available (because blood collection for serology was not performed or because sequencing was not performed); VOC: variant of concern.

*Outbreak 1* - The first outbreak occurred in a vessel that made an emergency stop at the port of Salvador on January 27, 2021 to provide medical care to one CM with clinical suspicion of COVID-19. Most (66.7%) of the 63 CM were Peruvian and the remaining (33.3%) were Spanish. In the following days, another six CM needed hospital medical assistance. According to the crew, every CM underwent RT-PCR for SARS-CoV-2 on January 7 and 12, 2021, and remained in quarantine before the vessel departed from the port of origin on January 14, 2021, when a rapid antigen test was also performed. All tests performed before boarding were negative. 

Nasopharyngeal swabs were collected on board for RT-PCR testing on January 27 and February 3, 4 and 10. Blood was collected on February 10, for serology. One CM had samples collected at the hospital. Over half (55.6%) of the crew was positive for SARS-CoV-2 by RT-PCR (Table II). Considering positive results for both RT-PCR and IgM, the positive rate for COVID-19 in this vessel was 60.3%. Most (76.3%) of the CM with laboratory evidence of infection developed clinical signs and symptoms (Table II). The amplified RNA fragments obtained from two RT-PCR-positive individuals were sequenced and the C.14 variant, which had not been detected in Brazil, was identified. 

*Outbreak 2* - The second outbreak occurred in a vessel that arrived in Salvador on March 31, 2021 (Table I). Most (81.8%) CM were Filipino, while 9.1% were Greek and 9.1% Ukrainian. On April 3, two CM complaining of severe headache and dyspnoea had clinical suspicion of COVID-19 and disembarked for hospitalisation. Two days later, another CM needed hospital medical assistance. 

On April 3, nasopharyngeal swabs for SARS-CoV-2 RT-PCR were collected from the 22 CM. In addition, nine CM had blood collected for serology on April 6, those who did not either refused blood collection (10 CM) or were hospitalised (three CM). RT-PCR was positive for SARS-CoV-2 in six (27.3%) individuals. One person tested positive for IgM and negative for RT-PCR - when considering this case, the attack rate of SARS-CoV-2 infection was 31.8%. The frequency of symptomatic infection was 57.1%. The RT-PCR-positive samples were sequenced and the P.1 variant of concern (VOC) of SARS-CoV-2 was identified in all of them. 

*Outbreak 3* - On April 11, 2021, a vessel whose crew was from Brazil (88.5%) and Peru (11.5%) arrived in Salvador (Table I). One CM disembarked for medical care after presenting weakness and low blood pressure. Given the suspicion of COVID-19, the entire crew was tested for SARS-CoV-2 through RT-PCR. The attack rate of SARS-CoV-2 infection was 84.6% and 68.2% of those with evidence of infection had symptoms (Table II).

*Outbreak 4* - On May 2, 2021, a vessel made an emergency stop at Salvador to provide medical care for two CM with clinical suspicion of COVID-19. On the same day, these CM were admitted to a hospital, where one received O_2_ support. Although they had clinical symptoms (Table II) and chest computed tomography scans compatible with COVID-19 (ground-glass opacity with up to 25% of parenchymal involvement), they tested negative by RT-PCR at the hospital. RT-PCR for SARS-CoV-2 was performed on samples from the remaining CM on board and two of them tested positive, despite being asymptomatic. Considering these RT-PCR results and the clinical and chest image findings suggestive of COVID-19 from two CM, the attack rate of SARS-CoV-2 infection in this vessel was 18.2% and half of the CM with evidence of infection had disease symptoms (Table II). 

*Outbreak 5* - The fifth outbreak was detected in a vessel anchored in Salvador on May 27, 2021. The vessel’s crew had four nationalities: 63% were Filipino, 18.5% Brazilian, 14.8% Greek and 3.7% Ukrainian. As it was reported that eight CM had flu-like symptoms, nasopharyngeal swabs for SARS-CoV-2 RT-PCR were collected from all CM on May 27, 2021. On June 1, 2021, three CM who had previously tested negative were re-tested. This vessel exhibited the highest SARS-CoV-2 infection rate, with 88.9% of the crew testing positive by RT-PCR. It also had the highest frequency of symptomatic infection (91.6%) (Table II). This was the first of the investigated outbreaks that had a CM vaccinated against COVID-19 (Table II).

*Outbreak 6* - On July 12, 2021, a vessel whose crew was Brazilian (96.3%) and Paraguayan (3.7%) anchored in Salvador with two people complaining of flu-like symptoms and having tested positive for COVID-19 in rapid tests carried out on board. On the same day, RT-PCR for SARS-CoV-2 was performed on the entire crew by a private laboratory. The two symptomatic CM and three other asymptomatic people tested positive. On July 13, RT-PCR was repeated by the state public health laboratory on all CM, confirming previous RT-PCR findings. 

One CM that was initially negative by RT-PCR was hospitalised with mild symptoms and discharged after one day. However, the next day the person returned to the hospital with worsened clinical symptoms and was admitted to the intensive care unit (ICU). RT-PCR testing was repeated and returned positive for SARS-CoV-2 infection. After 35 days of hospitalisation, the patient died. The SARS-CoV-2 infection attack rate in this vessel was 22.2%. One of the RT-PCR-positive samples was sequenced, and the VOC B.1.617.2 of SARS-CoV-2 was identified - a strain that had not been detected in the state of Bahia until then. 

*Outbreak 7* - The seventh outbreak occurred in a vessel with CM of seven nationalities: 48.4% were Filipino, 16.1% Indian, 12.9% Danish, 12.9% Polish, 3.2% Ukrainian, 3.2% British and 3.2% Spanish. Following the COVID-19 testing protocol on disembarkation, one CM was positive for SARS-CoV-2 by RT-PCR. Therefore, RT-PCR testing was repeated on the entire onboard crew on August 11 and 12, 2021, and three people, including the first positive individual, tested positive (all asymptomatic). The attack rate of SARS-CoV-2 infection in this vessel was 9.7%, and no symptomatic infection was observed (Table II). Previous use of at least one vaccine dose against COVID-19 was reported by 80.6% of the CM. One RT-PCR-positive sample was sequenced, and the VOC B.1.351 was identified, a strain that had not been detected in Bahia by then. 

*Outbreak 8* - On November 11, 2021, a vessel whose crew was from India (41.7%), Philippines (37.5%), Ukraine (8.3%), Croatia (8.3%) and Poland (4.2%) anchored in Salvador with a CM manifesting symptoms compatible with COVID-19. RT-PCR for SARS-CoV-2 was performed on the entire crew on the same day and again on November 18. On November 30, additional samples were collected from 14 CM who had previously tested negative. In the end, 10 CM were positive, representing a SARS-CoV-2 infection attack rate of 41.7% (Table II). Although this outbreak occurred in late 2021, when COVID-19 vaccines were already widely available, only 58.3% of the CM had been vaccinated with at least one dose of a COVID-19 vaccine. The RNAs obtained from four positive samples were sequenced, and the VOC B.1.617.2 (Delta) was identified in all of them.

## DISCUSSION

*Enclosed environments, vaccination and COVID-19 outbreaks* - The high attack rate (> 60%) of SARS-CoV-2 infections in three of the outbreaks (vessels 1, 3 and 4) well exemplify how enclosed environments favour the propagation of SARS-CoV-2 within confined populations. It also reflects the difficulty in implementing onboard measures to effectively prevent and control the spread of viruses like SARS-CoV-2, as seen in other cases.^8^ ^,^ ^9^ ^)^ For instance, adequate ventilation is critical in limiting the spread of the SARS-CoV-2 virus.^21^ ^,^ ^22^ However, improving ventilation in already built vessels is not always easy. The common spaces in carriers, tankers and cargo vessels are often confined and the cabins are shared between CM, as we observed on the vessels involved in the outbreaks we investigated. 

Besides the limited ventilation in closed vessel environments, several other factors may have influenced the high attack rates and frequency of symptomatic infections within each vessel, such as (i) the time elapsed between outbreak detection and control measures implementation - the earlier the vessel stops at a port after the suspicion/confirmation of infected cases, the better; here, it is likely that some vessels should have made an earlier emergency stop (in a previous port); (II) the strict implementation of all recommended control measures on board; and (iii) the strain of SARS-CoV-2 causing the outbreak, as some strains are more contagious or pathogenic than others. Some of these factors depend on the crew’s reality (e.g., if there are qualified health staff on board) and decisions (e.g., the captain’s decision to make an emergency stop) and on the shipping company’s support (appropriate training of staff, provision of tests and medications, among others) and rules. Other factors depend on the surveillance preparedness and available infrastructure of the place where the port is located. 

However, without diminishing the importance of all the factors cited above, our findings also suggest that the lack of vaccination against COVID-19 among CM may have been a crucial determinant for the SARS-CoV-2 infections and illness. We found that attack rates and the proportion of symptomatic infections tended to be higher in the early outbreaks that occurred before mid-2021 when crew use of a COVID-19 vaccine was non-existent. Furthermore, we found that prior use of at least one dose of a COVID-19 vaccine had an overall effectiveness of about 80% in preventing the development of the disease among those infected. Interpretation of this result should be cautious because our investigation was not designed to assess the effectiveness of COVID-19 vaccines, and we did not consider important vaccine-related factors, such as vaccine type, number of vaccine doses, time since the last dose and the different infectious variants of SARS-CoV-2. However, the protection rate that we observed was consistent with what most vaccine trials and observational studies have shown.^23^ ^,^ ^24^ ^)^ Furthermore, the protective effect of COVID-19 vaccination against the development of symptoms among those infected did not change after we adjusted the analysis for age. Thus, our findings are aligned with existing evidence on the importance of COVID-19 vaccination to prevent outbreaks among confined populations.^25^

Nevertheless, 10 CM who tested positive for SARS-CoV-2 had used a COVID-19 vaccine before traveling. This may have been due to partial protection because of an incomplete vaccination schedule or to the non-optimal time of traveling after completing the vaccination schedule. It is also possible that new viral strains could evade vaccine immunity. 

Most of the crew in the outbreaks reported here were young men with no history of comorbidities - yet symptomatic infections were frequently observed and severe and fatal cases occurred. These findings highlight the importance of policymakers emphasising the need for crew vaccination, including optimising sequential booster programs, especially for CM from countries with low vaccination coverage. The risk of unvaccinated individuals developing severe disease and even dying on the high seas can be high if the vessel cannot obtain the necessary aid on land.

*Challenges and combined control measures in vessels and at ports* - The COVID-19 pandemic has pushed policymakers and stakeholders to scrutinise international and local rules and regulations for dealing with an unprecedented global health emergency. It is essential that these guidelines orient a coordinated response among all the competent authorities involved, which often include a wide network of agencies, such as local, national, and international health surveillance offices, port operators and companies and operators of vessels from different nationalities.^26^ As indicated by the IHR, clearly defining the responsibilities of all the parties regarding the required measures, including isolation and quarantine, is critical for effective response.^17^

The measures adopted by some vessels before departure (e.g., testing and quarantine) were not enough to impede COVID-19 outbreaks from occurring, showing that they have limitations. The first investigated outbreak exemplifies this. Although tests were performed and quarantine among the unvaccinated crew was implemented before the vessel’s departure, 60% of the crew tested positive for SARS-CoV-2 after anchoring in Salvador. It is possible that the rapid antigen tests used for screening had low sensitivity and that people infected by then were not detected (false-negatives). Variable sensitivity among rapid SARS-CoV-2 antigen tests has been reported, with several commercial assays exhibiting suboptimal sensitivity.^27^ It is also possible that infected subjects were not in the optimal window for testing when RT-PCRs or rapid tests were performed before departure. Lastly, it is possible that all CM did not strictly follow the quarantine measures. Nevertheless, our investigation was not designed to assess the effectiveness of crew screening for SARS-CoV-2 infection before departure. In certain epidemiological scenarios of high SARS-CoV-2 transmission, crew screening with high sensitivity tests can be useful for reducing the risk of outbreaks on board. Further studies are needed to elucidate the role of SARS-CoV-2 screening. 

Although most CM cooperated with the investigations, in outbreak 2, a significant part of the crew refused blood collection for serology - this highlights the importance of building trust with the crew during such investigations. In all eight investigations, the tests carried out by the local surveillance and health laboratories increased the number of infected people detected on board. Notably, the early hospitalisations of the most severe cases, which may have prevented additional deaths, resulted from the joint efforts between the local health surveillance and the command of the vessels. Thus, trust and partnership between the surveillance team, the captain and the crew are crucial for successfully implementing actions to prevent the transmission of SARS-CoV-2 and provide proper medical care to patients. An epidemiological investigation of COVID-19 among 77 seafarers on an industrial ship in the Netherlands also emphasises how important is a partnership between the surveillance team, the captain and the crew during an outbreak.^14^

Another element that needs to be considered during vessel outbreaks is the extended time that vessels remain in port. For instance, while a vessel typically remains in the port of Salvador for 5 days, the mean number of days that the investigated vessels were anchored was 17 (range 10-25). This has economic and public health implications, including impacts on crew mental health, a significant issue for seafarers during the COVID-19 pandemic.^28^ ^,^ ^29^

Combining public health efforts between the crew and local health authorities is essential to reduce the transmission of SARS-CoV-2 within vessels and to contain the spread of the virus to port cities.^14^ ^,^ ^15^ The importance of these actions is reinforced by the discovery of three strains of SARS-CoV-2 that had not been detected in the state of Bahia before, indicating the role of maritime traffic in the spread of novel variants of the virus to new geographic regions if containment measures are not undertaken. Finally, our findings support the guidelines recommending that everyone traveling on vessels, whether crew or passengers, be vaccinated against COVID-19.

Some of the challenges identified during the investigations of COVID-19 outbreaks on vessels were language barriers, considering the diversity of the vessel’s flags and CM nationality, the need to convince the crew of the importance of participating in the investigation, and providing blood samples for testing (e.g., a significant number of CM was reluctant to give a blood sample on one of the vessels) and the ability to perform the timely diagnostic tests in the local laboratories, mainly due to the overload of the health system in some periods of the pandemic. All these issues should be seriously considered when IHR associated with maritime traffic are developed and revised.
